# *Mycobacterium abscessus* strain variability in preclinical drug development: does it really matter?

**DOI:** 10.1093/jac/dkae336

**Published:** 2024-09-23

**Authors:** Saskia E Mudde, Henriëtte M Meliefste, Nicole C Ammerman, Jurriaan E M de Steenwinkel, Hannelore I Bax

**Affiliations:** Department of Medical Microbiology and Infectious Diseases, Erasmus University Medical Center, Rotterdam, The Netherlands; Department of Medical Microbiology and Infectious Diseases, Erasmus University Medical Center, Rotterdam, The Netherlands; Department of Medical Microbiology and Infectious Diseases, Erasmus University Medical Center, Rotterdam, The Netherlands; Department of Medical Microbiology and Infectious Diseases, Erasmus University Medical Center, Rotterdam, The Netherlands; Department of Medical Microbiology and Infectious Diseases, Erasmus University Medical Center, Rotterdam, The Netherlands; Department of Internal Medicine, Section of Infectious Diseases, Erasmus University Medical Center, Rotterdam, The Netherlands

## Abstract

**Background:**

New treatment options for *Mycobacterium abscessus* infections are urgently needed. Since a correlation between MICs and clinical outcomes is not clearly established, potency of novel drugs needs to be evaluated using additional *in vitro* drug activity assays. Preclinical drug activity assays generally use the *M. abscessus* type strain ATCC 19977. However, *M. abscessus* complex entails a genetically and morphologically diverse group, and it is questionable whether drug activity observed against ATCC 19977 is representative of drug activity against clinical *M. abscessus* isolates.

**Objectives:**

To assess whether the relationship between MIC and the quantitative antimycobacterial activity of amikacin, imipenem and clofazimine differs between the ATCC 19977 strain and clinical *M. abscessus* isolates.

**Methods:**

Experiments were performed with *M. abscessus* ATCC 19977 and a subset of six clinical isolates covering the three *M. abscessus* subspecies and the smooth and rough morphotypes. Cultures were exposed to the drugs at 4-fold increasing, MIC-standardized concentrations, and the mycobacterial load was assessed over time.

**Results:**

Concentration- and time-dependent activity of amikacin, imipenem and clofazimine against the six clinical isolates was similar. Only slight variations in drug activity were observed between ATCC 19977 and clinical isolates.

**Conclusions:**

Time- and concentration-dependent drug activity against the ATCC 19977 strain seems indicative for *in vitro* drug behaviour against *M. abscessus* complex clinical isolates. Including one clinical smooth morphotype isolate alongside ATCC 19977 seems appropriate for reliable interpretation of this particular *in vitro* drug activity assay as part of the *M. abscessus* preclinical drug development pipeline.

## Introduction

*Mycobacterium abscessus* complex (MABC) comprises a group of rapidly growing non-tuberculous mycobacteria, notorious for their ability to cause detrimental lung infections, especially in patients with underlying lung disease.^1,2^ MABC’s intrinsic antibiotic resistance mechanisms severely complicate achieving successful treatment outcomes.^3^ Development and prioritization of promising drug candidates is essential to improve treatment success rates.

Determination of *in vitro* MICs is fundamental to antibiotic susceptibility testing, which can guide antibiotic treatment decisions. Yet, with MABC (and other non-tuberculous mycobacteria), the correlation between MICs and clinical treatment response is not clearly established.^4^ Potential factors involved in this uncertainty may include technical challenges, such as the impact of medium composition or drug instability,^5,6^ and discrepancies between *in vitro* settings (actively replicating, planktonic mycobacteria) and the complex host environment involving mycobacterial populations in different metabolic states and within biofilms. As such, MICs alone are insufficient for identifying promising new drugs and evaluating their clinical potential. Additional *in vitro* drug activity assays are needed to complement the preclinical drug development pipeline, such as time–kill kinetics assays (TKKs), which add value to endpoint measurements like MICs by assessing a drug’s inhibitory or killing activity over time.^7^

Apart from combining preclinical assays, including different isolates might be of additional interest when assessing the *in vitro* potential of novel drugs against *M. abscessus*. Currently, the type strain *M. abscessus* subsp. *abscessus* ATCC 19977 is most often used. However, being an environmental microorganism, MABC exhibits considerable genetic and phenotypic diversity, which potentially influences drug activity. Three genetically distinct MABC subspecies are distinguished (*abscessus*, *bolletii* and *massiliense*), as well as two different colony morphotypes (smooth and rough). This morphotype difference has been gaining interest over recent years, as rough isolates have been associated with increased virulence and poorer clinical outcomes than smooth isolates.^8–10^ Hence, the generalizability of *in vitro* preclinical drug activity testing based on ATCC 19977 alone could be questioned, and might benefit from a representative subset of clinical isolates being utilized. However, ‘a representative subset’ is not clearly defined, and the added value of including multiple isolates should first be investigated and balanced with practical feasibility.

Hence, this study assessed the variability in *in vitro* activity of amikacin, imipenem and clofazimine (three guideline-recommended antibiotics with distinct mechanisms of action^11^) against ATCC 19977 and six clinical MABC isolates with similar MICs. This subset covers the three subspecies and two morphotypes that MABC organisms can adopt as representatives of the diverse MABC family. TKKs were used to quantify concentration- and time-dependent activity of these cornerstone drugs across the different MABC clinical isolates. Thereby, this study aimed to assess whether the relationship between MICs and the quantitative antimycobacterial activity differed between clinical isolates and ATCC 19977. This investigation offers insight into the importance of incorporating diverse isolates into an important component of the preclinical development pipeline for MABC.

## Materials and methods

### Isolates, identification and culture conditions

Experiments included *M. abscessus* subsp. *abscessus* ATCC 19977 and six clinical MABC isolates. The clinical isolates were selected from the Erasmus MC clinical microbiology isolate collection based on colony morphotype, subspecies and susceptibility profile on record to obtain a representative set of isolates with similar MIC values for the three drugs assessed. Isolates were passaged up to four times on Mueller–Hinton agar [Becton, Dickinson, and Company (BD)] with 10% OADC (BD), followed by stock solution preparation in 7H9 Middlebrook broth (BD) with 10% OADC, 0.05% Tween80 and 0.5% glycerol. Stock solutions were stored at −80°C. Subspecies identification was confirmed by internal transcribed spacer (ITS) and *hsp65* sequencing (File S1, available as Supplementary data at *JAC* Online). In inconclusive cases, subsp. *massiliense* was differentiated from subsp. *abscessus/bolletii* based on *erm*(41) truncation (File S2). Isolate morphology was categorized as rough or smooth by visual inspection.

### Drug susceptibility testing

MICs of amikacin (Sigma–Aldrich), imipenem (Fresenius Kabi) and clofazimine (Sigma–Aldrich) were determined in duplicate according to the CLSI guidelines (M24, third edition)^4^ by broth microdilution in CAMHB (BD) at 30°C. Stock solutions of amikacin and imipenem were prepared in distilled water, and clofazimine in DMSO (Sigma–Aldrich).

### TKKs

Amikacin, imipenem and clofazimine activity against the MABC isolates was assessed using TKKs as previously described,^12^ with modifications regarding medium composition (no OADC) and culture volume (2.5 mL). Each isolate was cultured in CAMHB (±10^5^ bacterial/mL) and exposed to 4-fold increasing drug concentrations. Since drug MICs were similar, but not identical between the isolates, concentrations were based on isolate-specific MICs: ^1^/_4_ × , 1 × , 4 × , 16 ×  and additionally 64 ×  MIC for amikacin. For imipenem and clofazimine, 64 ×  MIC was excluded due to solubility issues. Cultures were incubated for 7 days at 30°C. Prior to the TKKs, drug stability of imipenem was assessed using the standard large-plate agar diffusion assay showing 40% daily decay, which was supplemented 50 µL/day to restore the original concentration. Amikacin and clofazimine were previously demonstrated to be stable.^12^ Cultures were sampled (100 µL) on multiple days for mycobacterial load determination. After 10-fold serial dilution, 3 × 10 µL per dilution was spotted onto Mueller–Hinton agar (BD) with 10% OADC, and 6 × 10 µL for undiluted samples, resulting in a lower limit of detection of 1.2 log_10_ cfu/mL. Cfu were counted after 4–7 days incubation at 30°C.

## Results and discussion

**Figure 1.**
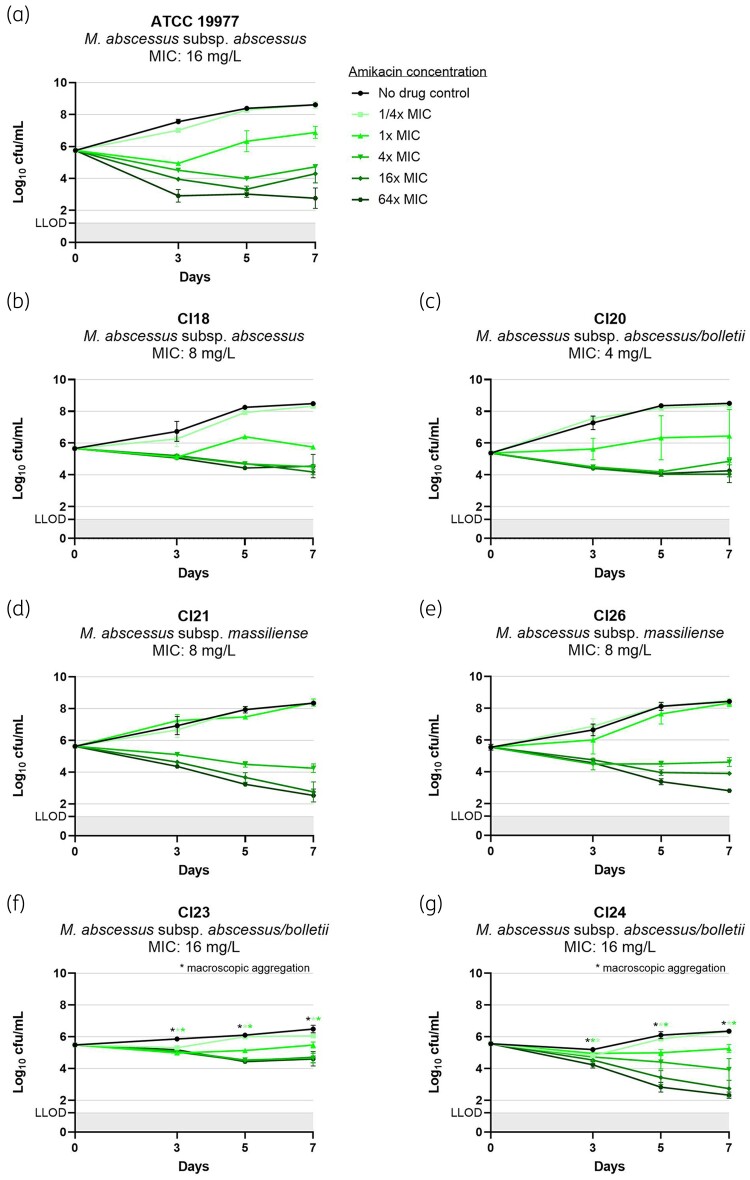
Time- and concentration-dependent activity of amikacin against seven MABC isolates: (a) ATCC 19977 (type strain): smooth morphotype *M. abscessus* subsp. *abscessus*; (b) Cl18: smooth morphotype *M. abscessus* subsp. *abscessus*; (c) Cl20: smooth morphotype *M. abscessus* subsp. *abscessus/bolletii*; (d) Cl21: smooth morphotype *M. abscessus* subsp. *massiliense*; (e) Cl26: smooth morphotype *M. abscessus* subsp. *massiliense*; (f) Cl23: rough morphotype *M. abscessus* subsp. *abscessus/bolletii*; (g) Cl24: rough morphotype *M. abscessus* subsp. *abscessus/bolletii*. Mycobacterial cultures were exposed to 4-fold increasing drug concentrations starting at ^1^/_4_ ×  the isolate-specific MIC. Experiments were performed in duplicate. Results are presented as mean log_10_ cfu/mL (± the range), with a lower limit of detection (LLOD) of 1.2 log_10_ cfu/mL. This figure appears in colour in the online version of *JAC* and in black and white in the print version of *JAC*.

**Figure 2.**
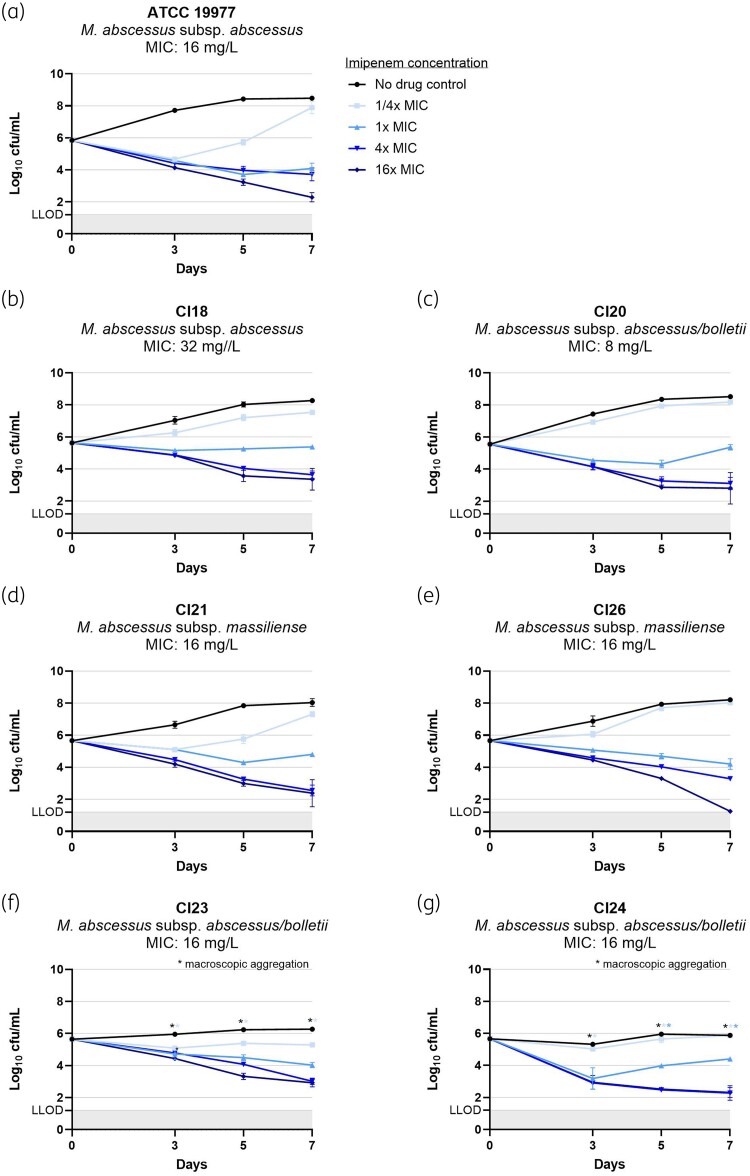
Time- and concentration-dependent activity of imipenem against seven MABC isolates: (a) ATCC 19977 (type strain): smooth morphotype *M. abscessus* subsp. *abscessus*; (b) Cl18: smooth morphotype *M. abscessus* subsp. *abscessus*; (c) Cl20: smooth morphotype *M. abscessus* subsp. *abscessus/bolletii*; (d) Cl21: smooth morphotype *M. abscessus* subsp. *massiliense*; (e) Cl26: smooth morphotype *M. abscessus* subsp. *massiliense*; (f) Cl23: rough morphotype *M. abscessus* subsp. *abscessus/bolletii*; (g) Cl24: rough morphotype *M. abscessus* subsp. *abscessus/bolletii*. Mycobacterial cultures were exposed to 4-fold increasing drug concentrations starting at ^1^/_4_ ×  the isolate-specific MIC. Experiments were performed in duplicate. Results are presented as mean log_10_ cfu/mL (± the range), with a lower limit of detection (LLOD) of 1.2 log_10_ cfu/mL. This figure appears in colour in the online version of *JAC* and in black and white in the print version of *JAC*.

The MABC clinical isolate collection included one smooth subsp. *abscessus* (Cl18), one smooth subsp. *abscessus/bolletii* (Cl20), two smooth subsp. *massiliense* (Cl21, Cl26) and two rough subsp. *abscessus/bolletii* (Cl23, Cl24). The MICs and TKK results for amikacin, imipenem and clofazimine per MABC isolate are displayed in Figure 1, Figure 2 and Figure S1, respectively. Of note, reliable cfu counting of the rough morphotypes was hampered by mycobacterial aggregation at high mycobacterial loads, as indicated in the figures.

Similar to ATCC 19977, amikacin at ^1^/_4_ ×  MIC was inactive against all clinical isolates. Activity of 1 ×  MIC against ATCC 19977 was inhibitory up to Day 3, followed by mycobacterial outgrowth, whereas against the clinical isolates, activity varied between being inactive or inhibitory. At 4–64 ×  MIC, amikacin demonstrated killing activity against all clinical isolates until at least Day 5, with the amount of killing being comparable for ATCC19977, Cl21, Cl26 and Cl24, and for Cl18, Cl20 and Cl23.

As for imipenem, compared with ATCC 19977, ^1^/_4_ ×  MIC demonstrated similar activity against Cl21, while little activity was observed against the other clinical isolates. Activity of 1 ×  MIC was somewhat variable among the isolates, demonstrating either slight outgrowth, stabilization or continued mycobacterial reduction after Day 3, whereas killing activity similar to ATCC 19977 was observed for 4 ×  and 16 ×  MIC against the clinical isolates.

Amongst the clinical isolates, clofazimine activity at ^1^/_4_ × –16 ×  MIC was comparable overall. However, activity of these concentrations was mostly inhibitory, whereas 1 ×  and 16 ×  MIC demonstrated killing activity instead against ATCC 19977. Of note, against ATCC 19977, activity of clofazimine at 1 ×  MIC lasted longer than 4 ×  MIC, which showed mycobacterial outgrowth after Day 3, thus 1 ×  MIC was more active than 4 ×  MIC. This observation has previously been described for other antimycobacterial drugs and has been attributed to the so-called Eagle effect: a paradoxical phenomenon where higher drug concentrations lead to less *in vitro* killing.^13,14^ The exact mechanisms responsible for this observation are unknown, although it has been speculated that high drug concentrations might inhibit RNA and protein synthesis necessary for killing.^15^ The relevance of this finding in the present study is debatable, particularly since this effect was less pronounced for the clinical isolates.

Macroscopic aggregation of the rough isolates posed a technical challenge in the TKKs, and likely led to an underestimation of the true cfu count, especially for the non-drug-exposed controls. Nevertheless, assuming that aggregation is related to high loads and that non-drug-exposed rough isolates exhibit a similar increase in cfu count over time compared with smooth isolates, the impact of different antibiotic concentrations on the mycobacterial load could still be assessed, and suggested similar overall activities of the three drugs between smooth and rough morphotypes. MABC morphotype is thought to be clinically relevant, as rough isolates have been associated with worse clinical outcomes.^9,10,16^ It is hypothesized that reduced levels of cell wall glycopeptidolipids in rough isolates expose virulence factors that are covered in smooth isolates.^8^ Such exposed virulence factors likely facilitate increased cording capabilities,^17^ which might contribute to the observed macroscopic aggregation in these experiments.

In conclusion, across a collection of six clinical isolates with similar MICs of amikacin, imipenem and clofazimine, and covering the different MABC subspecies and morphotypes, variability in concentration- and time-dependent activity was slight. Only slight variations were observed between both smooth and rough clinical isolates and ATCC 19977. Altogether, these findings indicate that drug activity over time against ATCC 19977 is generally suggestive of *in vitro* drug behaviour against other MABC isolates. Therefore, including one smooth clinical isolate alongside ATCC 19977 may be sufficient for reliable interpretation of this particular *in vitro* drug activity assay as part of the preclinical drug development pipeline. Considering the technical challenges as well as minimal variations in drug activity observed, excluding rough MABC isolates seems appropriate.

## Supplementary Material

dkae336_Supplementary_Data

## Supplementary data

Files S1 and S2 and Figure S1 are available as Supplementary data at *JAC* Online.

## References

[1] Qvist T , Taylor-Robinson D, Waldmann E et al Comparing the harmful effects of nontuberculous mycobacteria and Gram negative bacteria on lung function in patients with cystic fibrosis. J Cyst Fibros 2016; 15: 380–5. 10.1016/j.jcf.2015.09.007 26482717 PMC4893021

[2] Esther CR Jr , Esserman DA, Gilligan P et al Chronic *Mycobacterium abscessus* infection and lung function decline in cystic fibrosis. J Cyst Fibros 2010; 9: 117–23. 10.1016/j.jcf.2009.12.001 20071249 PMC3837580

[3] Johansen MD , Herrmann JL, Kremer L. Non-tuberculous mycobacteria and the rise of *Mycobacterium abscessus*. Nat Rev Microbiol 2020; 18: 392–407. 10.1038/s41579-020-0331-1 32086501

[4] CLSI . Susceptibility Testing of Mycobacteria, Nocardia spp., and Other Aerobic Actinomycetes—Third Edition: M24. 2018. 31339680

[5] Chapagain M , Pasipanodya JG, Athale S et al Omadacycline efficacy in the hollow fibre system model of pulmonary *Mycobacterium avium* complex and potency at clinically attainable doses. J Antimicrob Chemother 2022; 77: 1694–705. 10.1093/jac/dkac068 35257162 PMC9155607

[6] Schoutrop ELM , Brouwer MAE, Jenniskens JCA et al The stability of antimycobacterial drugs in media used for drug susceptibility testing. Diagn Microbiol Infect Dis 2018; 92: 305–8. 10.1016/j.diagmicrobio.2018.06.015 30025972

[7] Bax HI , Bakker-Woudenberg I, de Vogel CP et al The role of the time-kill kinetics assay as part of a preclinical modeling framework for assessing the activity of anti-tuberculosis drugs. Tuberculosis (Edinb) 2017; 105: 80–5. 10.1016/j.tube.2017.04.010 28610791

[8] Bernut A , Herrmann JL, Kissa K et al *Mycobacterium abscessus* cording prevents phagocytosis and promotes abscess formation. Proc Natl Acad Sci U S A 2014; 111: E943–52. 10.1073/pnas.1321390111 24567393 PMC3956181

[9] Hedin W , Fröberg G, Fredman K et al A rough colony morphology of *Mycobacterium abscessus* is associated with cavitary pulmonary disease and poor clinical outcome. J Infect Dis 2023; 227: 820–7. 10.1093/infdis/jiad007 36637124 PMC10043986

[10] Li B , Ye M, Zhao L et al Glycopeptidolipid genotype correlates with the severity of *Mycobacterium abscessus* lung disease. J Infect Dis 2020; 221: S257–62. 10.1093/infdis/jiz475 32176786

[11] Daley CL , Iaccarino JM, Lange C et al Treatment of nontuberculous mycobacterial pulmonary disease: an official ATS/ERS/ESCMID/IDSA clinical practice guideline. Clin Infect Dis 2020; 71: e1–36. 10.1093/cid/ciaa241 32628747 PMC7768748

[12] Mudde SE , Schildkraut JA, Ammerman NC et al Unraveling antibiotic resistance mechanisms in *Mycobacterium abscessus*: the potential role of efflux pumps. J Glob Antimicrob Resist 2022; 31: 345–52. 10.1016/j.jgar.2022.10.015 36347496

[13] Drlica K , Xu C, Wang JY et al Fluoroquinolone action in mycobacteria: similarity with effects in *Escherichia coli* and detection by cell lysate viscosity. Antimicrob Agents Chemother 1996; 40: 1594–9. 10.1128/AAC.40.7.1594 8807046 PMC163379

[14] de Knegt GJ , van der Meijden A, de Vogel CP et al Activity of moxifloxacin and linezolid against *Mycobacterium tuberculosis* in combination with potentiator drugs verapamil, timcodar, colistin and SQ109. Int J Antimicrob Agents 2017; 49: 302–7. 10.1016/j.ijantimicag.2016.11.027 28162983

[15] Prasetyoputri A , Jarrad AM, Cooper MA et al The Eagle effect and antibiotic-induced persistence: two sides of the same coin? Trends Microbiol 2019; 27: 339–54. 10.1016/j.tim.2018.10.007 30448198

[16] Catherinot E , Roux AL, Macheras E et al Acute respiratory failure involving an R variant of *Mycobacterium abscessus*. J Clin Microbiol 2009; 47: 271–4. 10.1128/JCM.01478-08 19020061 PMC2620830

[17] Howard ST , Rhoades E, Recht J et al Spontaneous reversion of *Mycobacterium abscessus* from a smooth to a rough morphotype is associated with reduced expression of glycopeptidolipid and reacquisition of an invasive phenotype. Microbiology (Reading) 2006; 152: 1581–90. 10.1099/mic.0.28625-0 16735722

