# Supplementary material for: Mycobacterium abscessus strain variability in preclinical drug development: does it really matter?
Source: J Antimicrob Chemother. 2024 Sep 23;79(12):3169–73. doi: 10.1093/jac/dkae336 (PMC11638096; doi:10.1093/jac/dkae336)
Supplement: dkae336_Supplementary_Data [file dkae336_supplementary_data.docx]

**Supplementary files**

**Supplementary file 1.** Subspecies identification by *ITS* and *hsp65* DNA sequencing.

Isolates were grown on Mueller Hinton agar plates (BD) supplemented with 10% OADC for 14 days prior to DNA isolation. After lysis in 250 µl MagNA Pure Bacteria Lysis Buffer (Roche) and heat shock inactivation (10 minutes, 95 °C), DNA was isolated on the MagNA Pure 96 platform using the Viral Nucleic Acid Small Volume kit (Roche) according to the manufacture’s Pathogen Universal Protocol. Per isolate, 5 µl DNA was combined with 12.5 µl 1x FastStart PR Master Mix (Roche), and 3 µM primers (see below). For the negative control, DNA was replaced by 5 µl dilution buffer. Cycling parameters involved pre-denaturation at 94 °C for 5 minutes, denaturation at 94 °C for 35 cycles of 30 seconds, annealing at 55 °C for 30 seconds, and extension at 72 °C for 1 minute. Gel electrophoresis using the E-gel Power Snap (Invitrogen) was used for PCR product confirmation. Amplicon sequences, obtained by BaseClear (Leiden, The Netherlands), were subjected to BLAST analysis using BioNumerics software (Applied Maths, Sint-Martens-Latem, Belgium). Subspecies were identified based on a ≥99% match with internal reference sequences.

Primers used for ITS and *hsp65* sequencing

| **Region** | **Primer** | **Sequence** |
| --- | --- | --- |
| ITS | MycoITS-F | 5’-ACCTCCTTTCTAAGGAGCACC-3’ |
| ITS | MycoITS-R | 5’-GATGCTCGCAACCACTATCCA-3’ |
| *hsp65* | Tb11 | 5’-ACCAACGATGGTGTGTCCAT-5’ |
| *hsp65* | Tb11 | 5’-CTTGTCGAACCGCATACCCT-3’ |

**Supplementary file 2.** Subspecies identification by *erm(41)* gene amplification and electrophoresis.

Thick solutions of each isolate were prepared in phosphate buffered saline, followed by centrifugation for 10 minutes at 21.000 xg. Supernatant was removed, and the pellet was suspended in 200 µl distilled water. After heat shock inactivation (10 minutes, 100 °C), samples were stored at -20 °C until further use. Per sample, a PCR mixture with a total volume of 25 µl was prepared, containing 2.5 µl bacterial suspension, 1x PCR taq buffer with (NH_4_)_2_SO_4_ (ThermoFisher), 2.5 mM MgCl_2_ (ThermoFisher), 0.1 mM dNTP’s (ThermoFisher), 0.008 U/µl Fermentas Taq-polymerase (ThermoFisher), 0.5 pmol/µl forward primer (5’-TCGCTCAGGGGAGTTCGTTG-3’), 0.5 pmol/µl reverse primer (5’-ATCACCAGCACCGACGAATC-3’), and distilled water. Cycling parameters were as follows: pre-denaturation at 94 °C for 2 minutes, denaturation at 94 °C for 30 cycles of 30 seconds, annealing at 55 °C for 30 seconds, extension at 72 °C for 30 seconds, and lastly post-elongation at 72 °C for 5 minutes. The PCR products, together with a GeneRuler 100 bp plus DNA-ladder (ThermoFisher) were loaded onto an 1%-agarose gel consisting of agarose (Sphaero) and 0.05% SYBR^TM^ Safe DNA Gel Stain (Invitrogen) in 0.5X TBE electrophoresis buffer (ThermoFisher). The gel was run for 30 minutes in 0.5X TBE electrophoresis buffer at 110 mA, and imaged using the Gel Doc System and Proxima AQ4 software (Isogen Life Science B.V.)


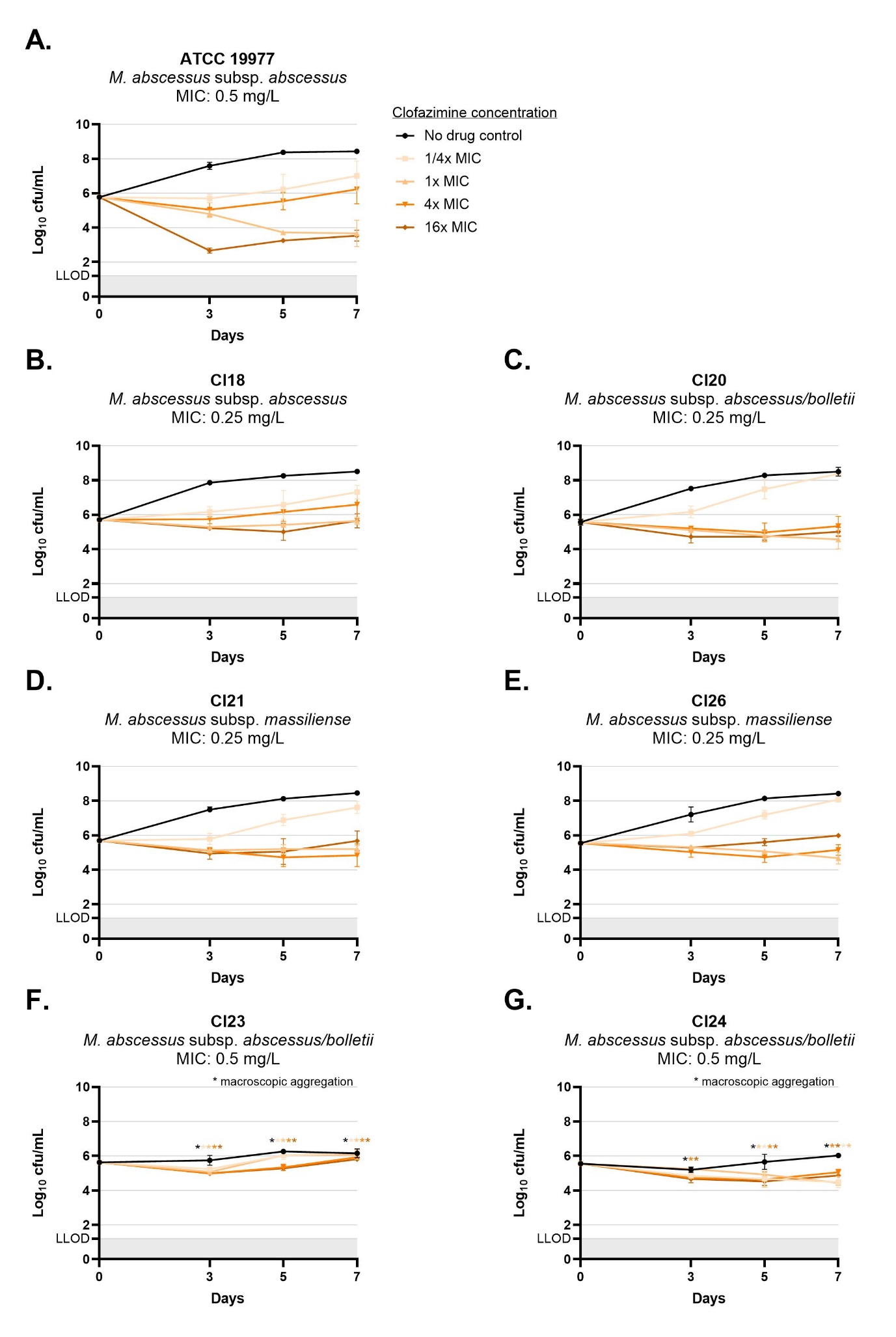


**Figure S1.** Time- and concentration dependent activity of clofazimine against 7 *Mycobacterium abscessus* complex isolates: A) ATCC 19977 (type strain): smooth morphotype *M. abscessus* subsp. *abscessus*; B) Cl18: smooth morphotype *M. abscessus* subsp. *abscessus*; C) Cl20: smooth morphotype *M. abscessus* subsp. *abscessus/bolletii*; D) Cl21: smooth morphotype *M. abscessus* subsp. *massiliense*; E) Cl26: smooth morphotype *M. abscessus* subsp. *massiliense*; F) Cl23: rough morphotype *M. abscessus* subsp. *abscessus/bolletii*; G) Cl24: rough morphotype *M. abscessus* subsp. *abscessus/bolletii*. Mycobacterial cultures were exposed to 4-fold increasing drug concentrations starting at 1/4x the isolate-specific MIC. Experiments were performed in duplicate. Results are presented as mean log_10_ colony forming units (cfu) per mL (+/- the range), with a lower limit of detection (LLOD) of 1.2 log_10_ cfu/mL. *Bacterial aggregation was visually present.
